# Rat Pial Microvascular Changes During Cerebral Blood Flow Decrease and Recovery: Effects of Cyanidin Administration

**DOI:** 10.3389/fphys.2018.00540

**Published:** 2018-05-15

**Authors:** Teresa Mastantuono, Martina Di Maro, Martina Chiurazzi, Laura Battiloro, Espedita Muscariello, Gilda Nasti, Noemy Starita, Antonio Colantuoni, Dominga Lapi

**Affiliations:** ^1^Department of Clinical Medicine and Surgery, “Federico II” University Medical School, Naples, Italy; ^2^Molecular Biology and Viral Oncology Unit, Istituto Nazionale Tumori IRCCS - “Fond. G. Pascale”, Naples, Italy

**Keywords:** cyanidin, cerebral blood flow reduction, reperfusion, pial microcirculation, reactive oxygen species, neuronal damage

## Abstract

The reactive oxygen species (ROS) are known to play a major role in many pathophysiological conditions, such as ischemia and reperfusion injury. The present study was aimed to evaluate the *in vivo* cyanidin (anthocyanin) effects on damages induced by rat pial microvascular hypoperfusion-reperfusion injury by cerebral blood flow decrease (CBFD) and subsequent cerebral blood flow recovery (CBFR). In particular, the main purpose was to detect changes in ROS production after cyanidin administration. Rat pial microvasculature was investigated using fluorescence microscopy through a cranial window (closed); Strahler's method was utilized to define the geometric features of pial vessels. ROS production was investigated *in vivo* by 2′-7′-dichlorofluorescein-diacetate assay and neuronal damage was measured on isolated brain sections by 2,3,5-triphenyltetrazolium chloride staining. After 30 min of CBFD, induced by bilateral common carotid artery occlusion, and 60 min of CBFR, rats showed decrease of arteriolar diameter and capillary perfusion; furthermore, increase in microvascular leakage and leukocyte adhesion was observed. Conversely, cyanidin administration induced dose-related arteriolar dilation, reduction in microvascular permeability as well as leukocyte adhesion when compared to animals subjected to restriction of cerebral blood flow; moreover, capillary perfusion was protected. ROS generation increase and marked neuronal damage were detected in animals subjected to CBFD and CBFR. On the other hand, cyanidin was able to reduce ROS generation and neuronal damage. In conclusion, cyanidin treatment showed dose-related protective effects on rat pial microcirculation during CBFD and subsequent CBFR, inducing arteriolar dilation by nitric oxide release and inhibiting ROS formation, consequently preserving the blood brain barrier integrity.

## Introduction

Many evidences indicate that a diet rich in antioxidants is associate to a decreased incidence of cardiovascular diseases, such as stroke, acute myocardial disease or cancer (Galvano et al., 2004; Lapi et al., 2016). Anthocyanins, belonging to polyphenol family, are one of the natural antioxidants responsible of fruit and flower colors (red, orange and blue) and play an important role in counteracting the oxidative stress induced by reactive oxygen species (ROS). These radicals have been related to different pathophysiological conditions (Serraino et al., 2003; Accetta et al., 2016; Mondola et al., 2016). Higher ROS production, indeed, appears to be associated to the pathogenesis of ischemia/reperfusion injury (Tsuda et al., 1999). On the other hand, the protective role of natural anthocyanins has been demonstrated to be effective against ischemia/reperfusion injury in different organs, such as kidney, heart and intestine (Jakesevic et al., 2013; Quintieri et al., 2013; Isaak et al., 2017). Moreover, we previously demonstrated the protective effects of Vaccinium myrtillus extract supplementation to the diet (containing 34.7% of anthocyanins) on hamster pial microcirculation during brain hypoperfusion-reperfusion. In particular, after 2, 4, and 6 months of oral supplementation, anthocyanins were able to counteract microvascular changes such as arteriolar vasoconstriction, increase of microvascular permeability and leukocyte adhesion (Mastantuono et al., 2016).

Cyanidin, belonging to the anthocyanin family, has been widely studied in *in vivo* and *in vitro* models (Galvano et al., 2004). In particular, Aguirre et al. and Fratantonio et al. observed that Cyanidin-3-O-glucoside presents many properties, such as anti-inflammatory and anti-tumor effects (Fratantonio et al., 2016; Olivas-Aguirre et al., 2016). Furthermore, this substance appears to enhance the release of nitric oxide (NO) and other vasodilating factors, thereby improving endothelial-dependent vasodilation (Sivasinprasasn et al., 2016). It is worth noting that ORAC (oxygen radical absorbance capacity) activity is highest for cyanidin compared to other anthocyanins (Zheng and Wang, 2003). Tsuda et al. have demonstrated that rats, treated with an orally administered Cyanidin-3-O-glucoside for 14 days, were preserved from hepatic I/R damage (Tsuda et al., 1999).

The aim of this study was to investigate the *in vivo* effects of cyanidin on oxidative stress and changes in rat pial microvasculature determined by 30 min of cerebral blood flow decrease (CBFD) and 60 min of cerebral blood flow recovery (CBFR). To do this, we evaluated ROS production during CBFD and CBFR, respectively, and assessed the antioxidant properties of cyanidin using 2′-7′-dichlorofluorescein-diacetate (DCFH-DA) assay. Finally, the neuronal damage was quantified by 2,3,5-triphenyltetrazolium chloride (TTC) staining.

## Materials and methods

### Experimental groups

Experiments were carried out utilizing male Wistar rats, 250–300g (Harlan, Italy), randomly assigned to three groups, as reported in Table 1: (1) sham group (SO group), subjected to the same surgical procedure of the other experimental groups without changes in cerebral blood flow; (2) reduced blood flow group (RF group) and (3) cyanidin-treated group (Cy group) underwent 30 min CBFD plus 60 min CBFR.

**Table 1:** Number of animals (N), experimental protocol and treatment of each group.

**Group**	**Subgroup**	**N**	**Experimental protocol**	**Treatment**
SO	SO-Na	14	Same surgical procedure as in the other experimental groups without changes in cerebral blood flow	saline solution, twice within 40 min interval
	SO-Cy_1_	5	Same surgical procedure as in the other experimental groups without changes in cerebral blood flow	10 mg/kg b.w. cyanidin, twice within 40 min interval
	SO-Cy_2_	5	Same surgical procedure as in the other experimental groups without changes in cerebral blood flow	20 mg/kg b.w. cyanidin, twice within 40 min interval
	SO-L	5	Same surgical procedure as in the other experimental groups without changes in cerebral blood flow	10 mg/kg b.w. L-NIO, twice within 40 min interval
RF		14	30 min CBFD plus 60 min CBFR	saline solution,10 min before CBFD and CBFR
Cy	Cy_1_	14	30 min CBFD plus 60 min CBFR	10 mg/kg b.w. cyanidin,10 min before CBFD and CBFR
	Cy_2_	14	30 min CBFD plus 60 min CBFR	20 mg/kg b.w. cyanidin,10 min before CBFD and CBFR
	L/Cy_2_	14	30 min CBFD plus 60 min CBFR	10 mg/kg b.w. L-NIO, prior to higher dosage cyanidin

SO animals were divided in subgroups: (a) SO-Na subgroup (*n* = 14) was injected with intravenous (i.v.) saline solution (0.9% NaCl); (b) SO-Cy subgroup (*n* = 10), successively divided in SO-Cy_1_ (*n* = 5) and SO-Cy_2_ (*n* = 5) subgroups, received i.v. cyanidin, 10 mg/kg body weight (b.w.) or 20 mg/kg b.w., respectively; (c) SO-L subgroup (*n* = 5) was infused with i.v. N5-(1-iminoethyl)-L-ornithine (L-NIO), 10 mg/kg b.w. The animals of each subgroup received the substances twice within 40 min interval.

RF group (*n* = 14) was injected with i.v. saline solution (0.9% NaCl), 10 min before the CBFD and at the beginning of CBFR.

Cy group was differentiated in the following subgroups: (a) Cy_1_ (*n* = 14) and Cy_2_ (*n* = 14), administered with i.v. cyanidin, 10 mg/kg b.w. or 20 mg/kg b.w., respectively, 10 min before the CBFD and at the beginning of CBFR; (b) L/Cy_2_ subgroup (*n* = 14) was administered with i.v. L-NIO, 10 mg/kg b.w., prior to i.v. higher dosage cyanidin (20 mg/kg b.w.).

Five animals for SO-Na subgroup, RF and Cy groups were investigated by *in vivo* fluorescence microscopy, to detect microcirculation damage; six rats were utilized to assess oxidative stress by DCFH-DA assay after CBFD (*n* = 3) and after CBFR (*n* = 3); in three animals tissue damage was evaluated by TTC staining. The rats belonging to the SO-Cy_2_ and SO-L subgroups were utilized only for microcirculation investigations.

### Drug administration

Each utilized drug (cyanidin or L-NIO) was dissolved in 0.5 mL saline solution and, successively, i.v. injected to rats within 3 min, 10 min before CBFD and at the beginning of CBFR.

We tested the effects of two cyanidin doses: 10 or 20 mg/kg b.w. Pilot experiments indicated that cyanidin dosages below 10 mg/kg b.w. were ineffective on the pial microvasculature; on the other hand, dosages higher than 20 mg/kg b.w. did not improve microvascular protection detected in the animals administered with 20 mg/kg b.w cyanidin before and after CBFD. In Table 2 we reported the data about animals treated with cyanidin at a dosage of 5 mg/kg or 30 mg/kg b.w (chosen as reference values) administered 10 min before CBFD and at the beginning of CBFR.

**Table 2:** Variations of the main parameters in the two pilot groups: Cy_A_ and Cy_B_ subgroup (rats treated with cyanidin at the doses of 5 mg/kg b.w. or 30 mg/kg b.w) and subjected to 30 min CBFR and 60 min CBFR, compared with RF group and Cy_2_ subgroup.

**Groups**	**Number of animals/arterioles (n)**	**Percent diameter changes (%)**		**Microvascular leakage (NGL)**	**Leukocyte adhesion (number of leukocyte/100 μm of venular length/30s)**	**Capillary perfusion (BFCL) (% reduction compared to baseline)**
		**After 30 min CBFD**	**After 60 min CBFR**	**After 60 min CBFR**		
RF group	5/25	85.0 ± 2.5^#^	75 ± 3^#^	0.48 ± 0.03^#^	10 ± 2^#^	48 ± 4^#^
Cy_A_ subgroup	5/25	90.5 ± 3.0^#^	80.0 ± 2.5^#^	0.40 ± 0.02^#^	8 ± 2^#^	40 ± 5^#^
Cy_B_ subgroup	5/25	134 ± 3.5^∧^	140 ± 4^∧^	0.24± 0.02^∧^	4 ± 1^∧^	12 ± 2^∧^
Cy_2_ subgroup	5/25	130 ± 3^∧^	138.0 ± 3.5^∧^	0.25 ± 0.03^∧^	5 ± 2^∧^	14 ± 3^∧^

Data are reported as Mean ± SEM;

# *p < 0.01 vs. Cy_A_ and Cy_2_ subgroup*,

∧ *p < 0.01 vs. RF group and Cy_B_ subgroup*.

Moreover, L-NIO, known to inhibit the NO release (Moreau et al., 1995; Lapi et al., 2012), was administered at the dosage of 10 mg/kg b.w., 10 min before i.v. infusion of higher dosage cyanidin (20 mg/kg b.w.). In pilot experiments L-NIO, 10 mg/kg b.w., was effective in blunting arteriolar dilation determined by i.v. injection of 10 mg/4 min L-arginine (diameter increase by 22.8 ± 2.0%, compared to basal values) or in abolishing vasodilation due to topical administration of 100 μM acetylcholine (diameter increase by 5.0 ± 1.5%, compared to basal values).

The protocol of drug administration was previously described (Lapi et al., 2016). Appropriately mixing 2′-7′-dichlorofluorescein-diacetate (DCFH-DA) and artificial cerebrospinal fluid (aCSF) allowed us to superfuse the pial layer with 250 mM DCFH-DA solution (Watanabe, 1998) for 30 min after CBFD. Sigma Chemical, St. Louis, MO, USA supplied all drugs.

### Rat preparation

All experiments conform to the Guide for the Care and Use of Laboratory Animals published by the US National Institutes of Health (NIH Publication No. 85-23, revised 1996) and to institutional rules for the care and handling of experimental animals, as previously reported (Lapi et al., 2012). The protocol was approved by the “Federico II” University Medical School of Naples, Ethical Committee (n° 2011/0059997, 24/05/2011).

Rats were anesthetized with intra peritoneal (i.p.) injection of α-chloralose, (60 mg/kg b.w. for induction; afterward 30 mg/kg b.w.) and mechanically ventilated after tracheotomy, according to the protocol previously reported (Lapi et al., 2012). Briefly, two catheters were placed, one in the right femoral artery and the other in the left femoral vein, respectively, for the measurement of arterial blood pressure and to inject the fluorescent tracers [fluorescein isothiocyanate bound to dextran, molecular weight 70 kDa (FD 70), 50 mg/100 g b.w., as 5% wt/vol solution in 3 min just once at the start of experiment after 30 min of the preparation stabilization; rhodamine 6G, 1 mg/100 g b.w. in 0.3 mL, as a bolus with supplemental injection throughout CBFD and CBFR (final volume 0.3 mL·100 g−1·h−1) to label leukocytes for adhesion evaluation]. Both carotid arteries were prepared for clamping.

Blood gases were measured on arterial blood samples at 30 min intervals (ABL5; Radiometer, Copenhagen, Denmark). The parameters monitored in all animals were: heart rate, mean arterial blood pressure, respiratory CO_2_ and blood gases values. They were stable within physiological ranges. Rectal temperature was recorded and maintained at 37.0 ± 0.5°C, as previously reported (Lapi et al., 2016).

The visualization of pial microvasculature was carried out as previously reported (Morii et al., 1986; Ngai et al., 1988; Lapi et al., 2012). Briefly, a closed cranial window was positioned at the level of the left frontoparietal cortex through an incision in the skin to operate a craniotomy. Cerebral cortex was preserved by overheating caused by drilling with saline solution superfusion of the skull. The dura mater was gently cut and displayed on the corner; a quarz microscope coverglass was bound to the skull bone. Artificial cerebrospinal fluid was superfused the cerebral surface with a rate of 0.5 mL/min. The composition of the aCSF was 119.0 mM NaCl, 2.5 mM KCl, 1.3 mM MgSO4•7H2O, 1.0 mM NaH2PO4, 26.2 mM NaHCO3, 2.5 mM CaCl2 and 11.0 mM glucose (equilibrated with 10.0% O2, 6.0% CO2 and 84.0% N2; pH 7.38 ± 0.02).

The decrease in cerebral blood flow (CBFD) was produced by clamping both common carotid arteries, previously prepared. The clamping was removed after 30 min; thereafter the pial microvasculature was investigated during the recovery of cerebral blood flow (CBFR), lasting 60 min (Hudetz et al., 1985).

### Fluorescence microscopy

A fluorescence microscope was utilized to study pial microvascular networks as previously described (Lapi et al., 2012). In brief, the microscope (Leitz Orthoplan, Wetzlar, Germany) was equipped with long-distance objectives (2.5 x, numerical aperture (NA) 0.08; 10 x, NA 0.20; 20 x, NA 0.25; 32 x, NA 0.40) a 10x eyepiece. Moreover, a x10 eyepiece and a filter block (Ploemopak, Leitz) were used. A 100-Watt mercury lamp was used for epiillumination with the corresponding filters for FITC and rhodamine 6G. A heat filter prevented overheating of the preparations (Leitz KG1). Pial microvascular networks were televised with a DAGE MTI 300 low-light level camera and stored through a computer-based frame grabber (Pinnacle DC 10 plus, Avid Technology, Burlington, MA, USA).

### Geometric detection of microvascular network

In each animal, first we characterized the arteriolar network by stop-frame images and pial arterioles were assigned order according to Strahler's method, starting from capillaries to the largest arterioles (centripetal method), as previously reported (Kassab et al., 1993; Lapi et al., 2008). In each experiment we studied one order 4 arteriole, two order 3 and two order 2 arterioles. Furthermore, we assessed the functional changes of each arteriolar order under the experimental conditions. We report, however, the results detected in order 2 arterioles.

### Microvascular parameter assessment

Microvascular parameters were measured off-line utilizing a computerized imaging technique, previously described in details by Lapi et al. (2012) and Lapi et al. (2016). Concisely, arteriolar diameters were measured with a computerized method, Microvascular Imaging Program (MIP), frame by frame. The increase in permeability was measured by evaluating fluorescent dextran extravasation from venules and expressed as normalized gray levels (NGL): NGL = (I – Ir)/Ir, where Ir is the baseline gray level at the microvasculature filling with fluorescence, and I is the value at the end of CBFD or CBFR. Gray levels were obtained using the MIP image program by average of 5 windows, measuring 50 × 50 mM (10x objective) and located outside the venules. During recordings the same regions of interest were localized by a computer-assisted device for XY movement of the microscope table.

Leukocytes sticking to the vessel walls (45 venules for every group) over a 30-s time-period were reported as number of adherent cells/100 μm of venular length (v.l.)/30 s, utilizing appropriate magnification (20 x and 32 x, objectives) (Lapi et al., 2012). Perfused capillaries were evaluated as the length of the capillaries showing blood flow (BFCL), assessed by MIP image in an area of 150 × 150 μm (Lapi et al., 2016).

A Gould Windograf recorder (model 13-6615-10S, Gould, OH, USA) was utilized to record arterial blood pressure (mean), by Viggo-Spectramed P10E2 transducer; Oxnard, CA, USA, linked to catheterized femoral artery, and heart rate, as previously reported (Lapi et al., 2012). We measured the arterial blood gases (ABL5; Radiometer, Copenhagen, Denmark) at 30 min intervals, as previously reported (Lapi et al., 2012), as well as the hematocrit in basal conditions, at the end of CBFD and CBFR.

### ROS production evaluation

Superfusion of the pial layer with artificial cerebrospinal fluid, containing 250 mM 2′-7′-dichlorofluorescein-diacetate (DCFH-DA) at 37.0 ± 0.5°, was carried out after 30 min CBFD (*n* = 3) or 60 min CBFR (*n* = 3), as previously reported (Lapi et al., 2013). DCFH-DA is widely used as a marker for oxidative stress of the cells and tissues (Wang and Joseph, 1999). DCF fluorescence intensity, related to the intracellular ROS level, was assessed using an appropriate filter (522 nm) and measured by NGL (Watanabe, 1998).

### Tissue damage estimation

At the end of CBFR, rats were sacrificed to evaluate tissue damage. The brains were isolated and rostro-caudally cut into coronal sections (1 mm) with a vibratome (Campden Instrument, 752 M; Lafayette, IN, USA). Slices were incubated in 2% 2,3,5-triphenyltetrazolium chloride (TTC) (20 min) at 37°C and in 10% formalin overnight, as previously reported (Lapi et al., 2013). TTC, a white salt, is reduced to red 1,3,5-triphenylformazan by dehydrogenases in living cells. The location and extent of necrotic areas were assessed by computerized image analysis (Image-Pro Plus; Rockville, MD, USA). Moreover, the infarct size was quantified by manual measurements, according to the following formula: [(area of nonhypoperfused, or area not subjected to cerebral blood flow decrease, cortex or striatum – area of remaining hypoperfused, or area subjected to cerebral blood flow decrease, cortex or striatum)/area of nonhypoprfused cortex or striatum] × 100 (Bederson et al., 1986).

### Statistical analysis

All data were reported as mean ± SEM. Normal distribution of data was assessed with the Kolmogorov-Smirnov test. Parametric (Student's *t*-tests, ANOVA and Bonferroni post hoc test) or nonparametric tests (Wilcoxon, Mann-Whitney and Kruskal-Wallis tests) were utilized, according to data distribution; diameter and length data among experimental groups were compared with nonparametric tests, as previously reported (Lapi et al., 2016). Data derived from DCFH-DA treated rats were analyzed with non-parametric tests. SPSS 14.0 statistical package (IBM Italia, Segrate, MI, Italy) was used. Statistical significance was set at *p* < 0.05.

## Results

Under baseline conditions, Sthraler's method was used to differentiate arterioles in pial microvascular networks of all animals according to a centripetal scheme (Lapi et al., 2008). In particular, five orders of arterioles were observed, assigning order 5 to the largest vessels (mean diameter 62.6 ± 4.5 μm) up to the smallest ones, identified as order 1 (mean diameter: 15.8 ± 2.0 μm). Order 0 was assigned to the capillaries, sprouting from order 1 arterioles.

### SO group

**Figure 1:**
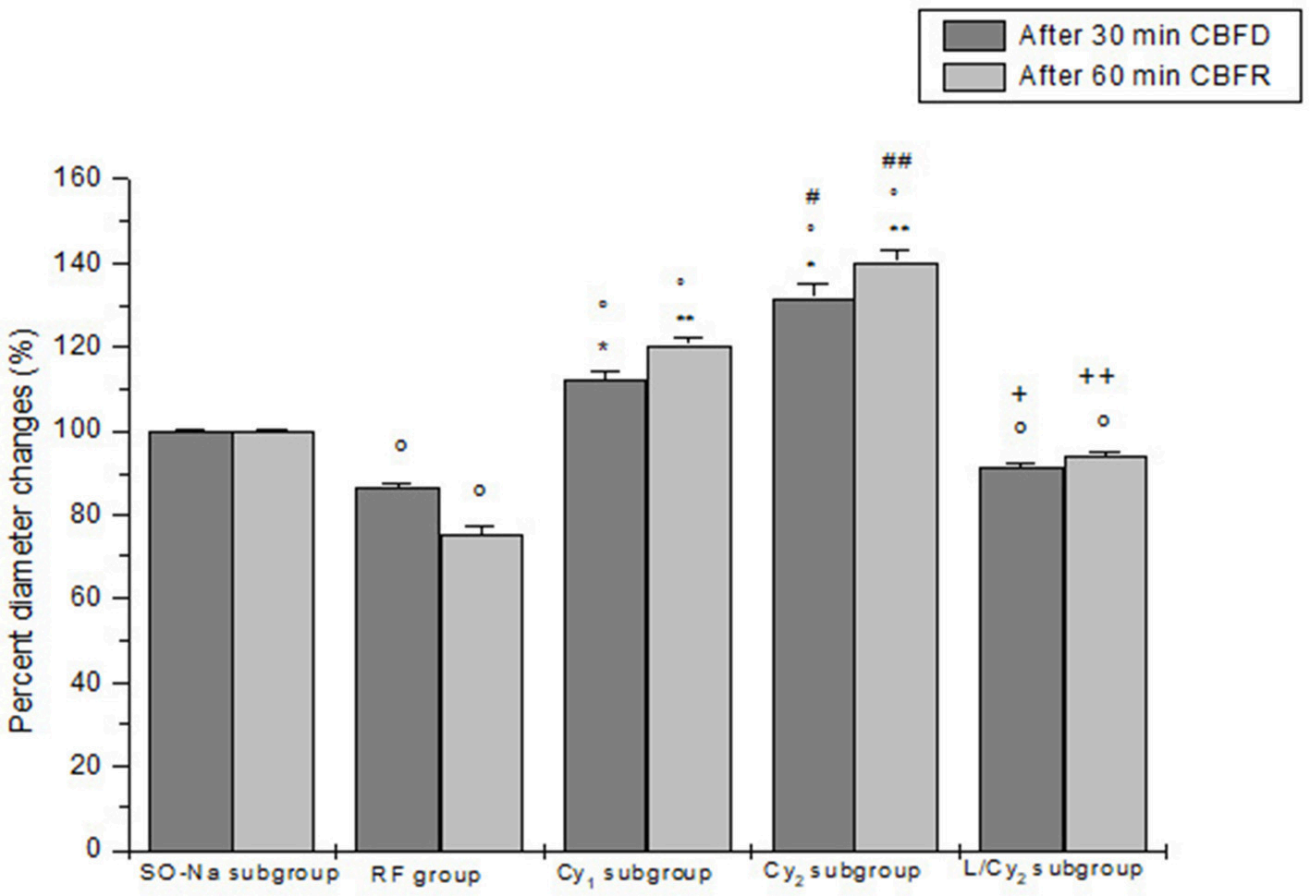
Diameter changes of order 2 arterioles, expressed in percent of baseline after 30 min cerebral blood flow reduction (CBFD) and after 60 min cerebral blood flow recovery (CBFR), in SO-Na subgroup (SO-Na), reduced blood flow group (RF), lower dosage cyanidin-treated subgroup (Cy_1_), higher dosage cyanidin-treated subgroup (Cy_2_) and higher dosage cyanidin plus L-NIO-treated subgroup (L/Cy_2_). Data are reported as Mean ± SEM. °*p* < 0.01 vs. SO-Na subgroup; ^*^p < 0.01 vs. RF group after 30 min CBFD; ^**^p < 0.01 vs. RF group after 60 min CBFR; ^+^*p* < 0.01 vs. Cy_2_ subgroup after 30 min CBFD; ^++^*p* < 0.01 vs. Cy_2_ subgroup after 60 min CBFR; ^#^*p* < 0.01 vs. Cy_1_ subgroup after 30 min CBFD; ^##^*p* < 0.01 vs. Cy_1_ subgroup after 60 min CBFR.

After the investigation period, no differences in microvascular parameters were detected in SO-Na subgroup, as reported in Table 3 and Figure 1. Furthermore, DCF fluorescence intensity did not change (0.04 ± 0.02 NGL) in the animals belonging to the SO-Na subgroup, superfused with DCFH-DA (Figure 3A).

**Table 3:** Variations of the main parameters at the end of reperfusion in SO-Na subgroup (SO-Na), reduced blood flow group (RF), lower dosage cyanidin-treated subgroup (Cy_1_), higher dosage cyanidin-treated subgroup (Cy_2_) and higher dosage cyanidin plus L-NIO-treated subgroup (L/Cy_2_).

**Groups**	**Number of animals/arterioles (n)**	**Microvascular leakage (NGL)**	**Leukocyte adhesion (number of leukocyte/100 μm of venular length/30 s)**	**Capillary perfusion (BFCL) (% reduction compared to baseline)**
SO-Na subgroup	5/25	0.02 ± 0.01	2 ± 1	0 ± 5
RF group	5/25	0.48 ± 0.03^§^^°^	10 ± 2^§^^°^	48 ± 4^§^^°^
Cy_1_ subgroup	5/25	0.36 ± 0.02^§^^°^^*^	7 ± 1^§^^°^^*^	22 ± 6^§^^°^^*^
Cy_2_ subgroup	5/25	0.25 ± 0.03^§^^°^^*^	5 ± 2^*^	14 ± 3^§^^°^^*^
L/Cy_2_ subgroup	5/25	0.27 ± 0.02^§^^°^^*^	6 ± 1^*^	17 ± 5^§^^°^^*^

Leukocyte adhesion: n = 45 venules for each entry. Data are reported as Mean ± SEM;

§ p < 0.01 vs. baseline;

° p < 0.01 vs. SO-Na subgroup;

* *p < 0.01 vs. RF group*.

Cyanidin injection caused arteriolar dilation in dose-related manner: order 2 arteriole diameter increased by 15.1 ± 1.8 and 34.6 ± 2.4% of baseline (*p* < 0.01 vs. baseline) in the rats of SO-Cy_1_ and SO-Cy_2_ subgroups, respectively. However, no significant changes in the other microvascular parameters and DCF fluorescence intensity were observed. Furthermore, after L-NIO injection, no significant variations of all parameters were detected in the animals of the SO-L subgroup.

### RF group

The decrease in cerebral blood flow for 30 min was accompanied by a reduction in diameter of all arteriolar orders with a decrease by 13.8 ± 1.5% of baseline in order 2 arterioles (*p* < 0.01 vs. baseline: mean diameter 25.6 ± 2.2 μm, and SO-Na subgroup; Figure 1). Leakage of fluorescent dextran was detected along the venules, indicating increased microvascular permeability (0.27 ± 0.02 NGL; *p* < 0.01 vs. baseline and SO-Na subgroup). Furthermore, fluorescence intensity increase was observed in animals subjected to DCFH-DA superfusion, demonstrating an increased ROS generation (Figure 3A).

**Figure 2:**
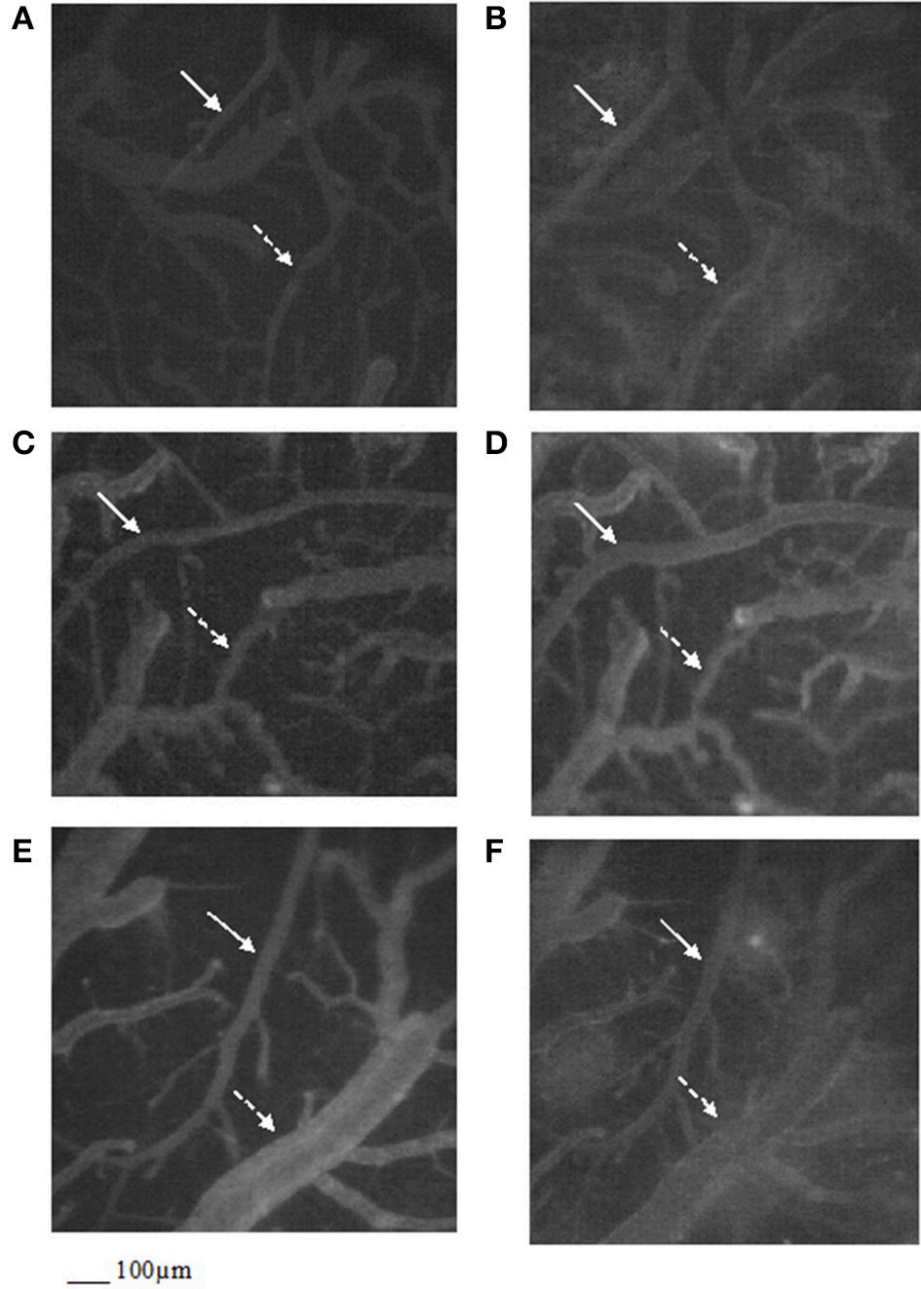
Computer-assisted images of the pial microvascular network under baseline conditions **(A)** and after cerebral blood flow decrease and recovery **(B)** in a rat of RF group: the increase in microvascular leakage is outlined by the marked change in the color of interstitium (from black to white). Computer-assisted images of the pial microvascular network under baseline conditions **(C)** and after cerebral blood flow decrease and recovery **(D)** in a higher dosage cyanidin-treated animal: no leakage of fluorescent-dextran was detected. Computer-assisted images of the pial microvascular network under baseline conditions **(E)** and after cerebral blood flow decrease and recovery **(F)** in a higher dosage cyanidin-treated plus L-NIO-treated rat. The arterioles are indicated by the white arrows, while the venules are outlined by dashed white arrows.

**Figure 3:**
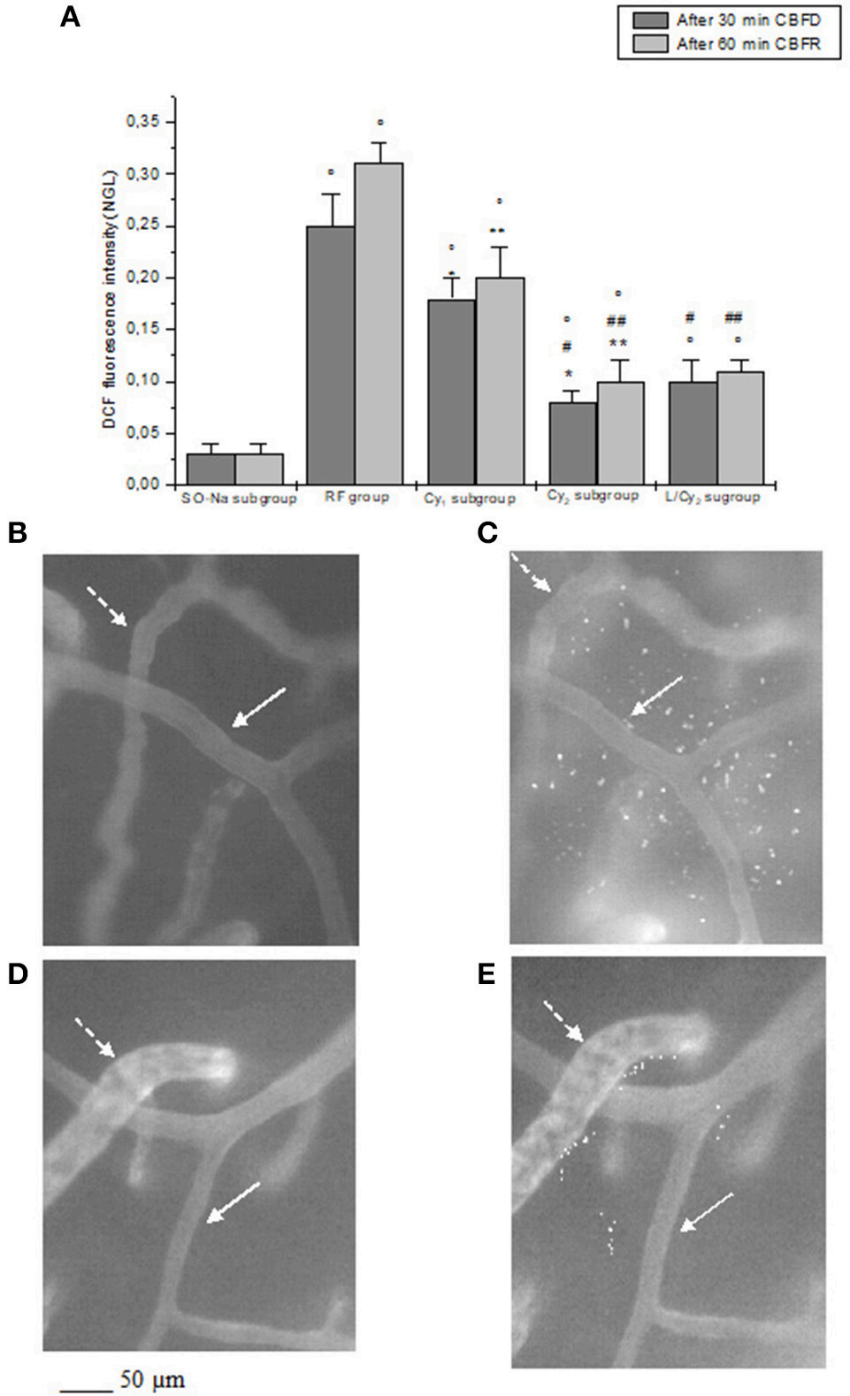
DCF fluorescence intensity after 30 min cerebral blood flow reduction (CBFD) and after 60 min cerebral blood flow recovery (CBFR) in the experimental groups **(A)**: SO-Na subgroup (SO-Na), reduced blood flow group (RF), lower dosage cyanidin-treated subgroup (Cy_1_), higher dosage cyanidin-treated subgroup (Cy_2_) and higher dosage cyanidin plus L-NIO-treated subgroup (L/Cy_2_). Data are reported as Mean ± SEM. °*p* < 0.01 vs. SO-Na subgroup; ^*^p < 0.01 vs. RF group after 30 min CBFD; ^**^p < 0.01 vs. RF group after 60 min CBFR; ^+^*p* < 0.01 vs. Cy_2_ subgroup after 30 min CBFD; ^++^*p* < 0.01 vs. Cy_2_ subgroup after 60 min CBFR; ^#^*p* < 0.01 vs. Cy_1_ subgroup after 30 min CBFD; ^##^*p* < 0.01 vs. Cy_1_ subgroup after 60 min CBFR. Computer-assisted images of the pial microvascular network under baseline conditions **(B)** and after cerebral blood flow decrease and recovery **(C)** in a rat of RF group: several fluorescent spots are outlined by the marked change in the color of interstitium (from black to white). Computer-assisted images of the pial microvascular network under baseline conditions **(D)** and after cerebral blood flow decrease and recovery **(E)** in a higher dosage cyanidin-treated animal: decreased number of fluorescent spots are detected. The arterioles are indicated by the white arrows, while the venules are outlined by dashed white arrows.

At the end of CBFR, all arteriolar orders presented a decrease in diameter when compared to baseline. Order 2 arteriole diameter diminished by 24.5 ± 2.0% of baseline (*p* < 0.01 vs. baseline and SO-Na subgroup; Figure 1). Moreover, dextran leakage significantly increased indicating marked microvascular permeability (0.48 ± 0.03 NGL; *p* < 0.01 vs. baseline and SO-Na subgroup); there was also increase in leukocytes adhesion (10 ± 2/100 μm v.l./30 s; *p* < 0.01 vs. baseline and SO-Na subgroup; Figures 2A,B). BFCL was reduced by 48.0 ± 4.0% of baseline (*p* < 0.01 vs. baseline and SO-Na subgroup) (Table 3). Finally, DCF fluorescence intensity was marked in the rats subjected to DCFH-DA perfusion, indicating a further increase in ROS formation: NGL were 0.31 ± 0.02 (*p* < 0.01 vs. SO-Na subgroup; Figures 3A–C).

### Cy group

Cyanidin, at 10 mg/kg b.w. dosage (Cy_1_ subgroup), prevented damage of the microvascular networks, inducing an increase in arteriolar diameter at the end of CBFD. In particular, order 2 arterioles dilated by 12.5 ± 1.8% of baseline (*p* < 0.01 vs. baseline: mean diameter 25.2 ± 2.3 μm, SO-Na subgroup and RF group; Figure 1). Microvascular permeability significantly decreased compared to RF group (0.22 ± 0.03 NGL; *p* < 0.01 vs. baseline, SO-Na subgroup and RF group) as well as DCF fluorescence intensity (0.18 ± 0.02 NGL; Figure 3A) (*p* < 0.01 vs. baseline, SO-Na subgroup and RF group).

After 60 min CBFR, all pial arterioles dilated: order 2 arteriole diameter increased by 20.5 ± 1.5% of baseline (*p* < 0.01 vs. baseline, SO-Na subgroup and RF group; Figure 1). Furthermore, fluorescent dextran leakage was significantly reduced compared to RF group (0.36 ± 0.02 NGL; *p* < 0.01 vs. baseline, SO-Na subgroup and RF group) as well as leukocytes adhering to venular walls (7 ± 1/100 μm v.l./30 s; *p* < 0.01 vs. baseline, SO-Na subgroup and RF group). BFCL decreased by 22.0 ± 2.2% of baseline (*p* < 0.01 vs. baseline, SO-Na subgroup and RF group) (Table 3). Finally, a slight increase in DCF fluorescence intensity was observed in rats belonging to the Cy_1_ subgroup, after DCFH-DA superfusion: NGL were 0.20 ± 0.03 (*p* < 0.01 vs. baseline, SO-Na subgroup and RF group) (Figure 3A).

Cyanidin, at the dosage 20 mg/kg b.w. (Cy_2_ subgroup), determined a marked dilation in all arterioles compared to Cy_1_ subgroup at the end of CBFD. There was an increase in order 2 arteriole diameter by 31.7 ± 3.0% of baseline (*p* < 0.01 vs. baseline: mean diameter 27.0 ±1.5 μm, SO-Na subgroup, RF group and Cy_1_ subgroup; Figure 1). Microvascular leakage appeared to be blunted compared to animals of RF and Cy_1_ subgroup (0.16 ± 0.02 NGL; *p* < 0.01 vs. baseline, SO-Na subgroup, RF group and Cy_1_ subgroup). Intensity in DCF fluorescence appeared to be slight (0.08 ± 0.01 NGL; *p* < 0.01 vs. baseline, SO-Na subgroup, RF group and Cy_1_ subgroup) (Figure 3A).

After 60 min CBFR, order 2 arteriole diameter increased up to 40.5 ± 2.8% of baseline (*p* < 0.01 vs. baseline, SO-Na subgroup, RF group and Cy_1_ subgroup; Figure 1). Additionally, there was a significant decrease in fluorescent dextran leakage when compared to RF and Cy_1_ subgroup (0.25 ± 0.03 NGL; *p* < 0.01 vs. baseline, SO-Na subgroup, RF group and Cy_1_ subgroup). Leukocyte adherent to venules were 5 ± 2/100 μm v.l./30 s, while BFCL decreased by 14.0 ± 2.0% of baseline (*p* < 0.01 vs. baseline, SO-Na subgroup, RF group and Cy_1_ subgroup) (Table 3 and Figures 2C,D). Finally, ROS production was completely counteracted by higher dosage cyanidin administration, as detected by reduction in fluorescence intensity in Cy_2_ subgroup, superfused with DCFH-DA: NGL were 0.10 ± 0.02 (*p* < 0.01 vs. baseline, SO-Na subgroup, RF group and Cy_1_ subgroup) (Figures 3A,D,E).

In L/Cy_2_ subgroup, L-NIO injection (10 mg/kg b.w.) 10 min before cyanidin treatment at the higher dose, blunted cyanidin effects on arteriolar diameter within 30 min of CBFD. In particular, diameter of order 2 arterioles was reduced by 8.7 ± 1.2% of baseline (mean diameter: 24.3 ± 1.7 μm; *p* < 0.01 vs. Cy_2_ subgroup; Figure 1). Conversely, L-NIO administration did not alter cyanidin effects on microvascular permeability (Table 3).

At the end of CBFR, all order 2 pial arterioles constricted with a decrease by 6.2 ± 1.0% of baseline diameter (*p* < 0.01 vs. Cy_2_ subgroup; Figure 1). However, microvascular permeability and adhesion of leukocytes were not influenced by L-NIO administration (Figures 2E,F). BFCL decreased by 17.0 ± 1.5% of baseline (*p* < 0.01 vs. baseline, SO-Na and RF groups; Table 3). No differences in DCF fluorescence were observed in L/Cy_2_ subgroup when compared to Cy_2_ subgroup animals: NGL were 0.10 ± 0.02 and 0.11 ± 0.01 at 30 min CBFD and 60 min CBFR, respectively (*p* < 0.01 vs. Rf group; Figure 3A).

No significant changes in MABP, heart rate, respiratory CO_2_ and blood gases were detected among the different subgroups under baseline conditions, at the end of CBFD and CBFR (as reported in Table 4).

Table 4(A) Mean arterial blood pressure (MABP), heart rate, respiratory CO_2_ and (B) blood gases (pCO_2_ and pO_2_) under baseline conditions, at the end of CBFD and CBFR in all subgroups.

**Table:** 

**Groups**	**MAPB (mmHg)**			**Heart rate (bpm)**			**Respiratory CO**_**2**_ **(mmHg)**		
	**Baseline**	**CBFD**	**CBFR**	**Baseline**	**CBFD**	**CBFR**	**Baseline**	**CBFD**	**CBFR**
**A**									
SO-Na	100.0 ± 1.5	–	–	328.5 ± 1.8	–	–	30.5 ± 1.5	–	–
SO-Cy_1_	101.0 ± 1.6	–	–	330.0 ± 1.5	–	–	30.8 ± 1.6	–	–
SO-Cy_2_	101.0 ± 1.4	–	–	325.0 ± 1.3	–	–	30.6 ± 1.8	–	–
SO-L	102.0 ± 1.5	–	–	327.0 ± 1.9	–	–	29.8 ± 2.0	–	–
RF	101.0 ± 1.7	92.5 ± 1.6^*^	97.5 ± 1.8	320.0 ± 1.5	315.5 ± 1.7	324.0 ± 2.0	30.2 ± 1.8	29.3± 1.5	30.8 ± 1.4
Cy_1_	102.0 ± 1.8	93.7 ± 1.5^*^	99.7 ± 1.6	321.5 ± 1.7	318.0 ± 1.5	325.0 ± 2.0	30.5 ± 1.5	30.5 ± 1.4	31.0 ± 1.6
Cy_2_	104.0 ± 2.8	95.8 ± 1.4^*^	100.0 ± 2.5	328.0 ± 1.6	320.5 ± 1.8	330.0 ± 1.5	30.6 ± 1.8	30.5 ± 1.7	30.9 ± 1.5
L/Cy_2_	106.0 ± 2.5	97.5 ± 1.6^*^	101.5 ± 2.0	320.8 ± 1.8	318.0 ± 1.4	320 ± 2.5	30.8 ± 2.0	31.0 ± 1.7	30.8 ± 1.8

**Table:** 

**Groups**	**pCO**_2_ **(mmHg)**			**pO**_2_ **(mmHg)**		
	**Baseline**	**CBFD**	**CBFR**	**Baseline**	**CBFD**	**CBFR**
**B**							
SO-Na	41.5 ± 2.0	–	–	96.5 ± 2.0	–	–
SO-Cy_1_	41.8 ± 1.8	–	–	97.0 ± 1.8	–	–
SO-Cy_2_	41.0 ± 1.5	–	–	97.2 ± 1.7	–	–
SO-L	42.0 ± 1.6	–	–	96.0 ± 2.2	–	–
RF	40.2 ± 2.1	40.1 ± 1.8	40.1 ± 2.1	96.8 ± 2.0	96.1 ± 1.8	96.0 ± 1.8
Cy_1_	40.8 ± 1.7	40.8 ± 1.6	40.5 ± 1.5	97.1 ± 1.7	98.0 ± 1.8	98.0 ± 1.5
Cy_2_	40.6 ± 2.0	41.0 ± 1.9	40.9 ± 1.7	97.0 ± 1.8	97.5 ± 1.7	97.0 ± 2.0
L/Cy_2_	40.8 ± 2.2	40.5 ± 2.0	40.4 ± 1.9	96.4 ± 2.1	96.0 ± 1.9	96.0 ± 1.7

Data are reported as Mean ± SEM;

* *p < 0.01 vs. baseline*.

### 2,3,5-triphenyltetrazolium chloride (TTC) staining

**Figure 4:**
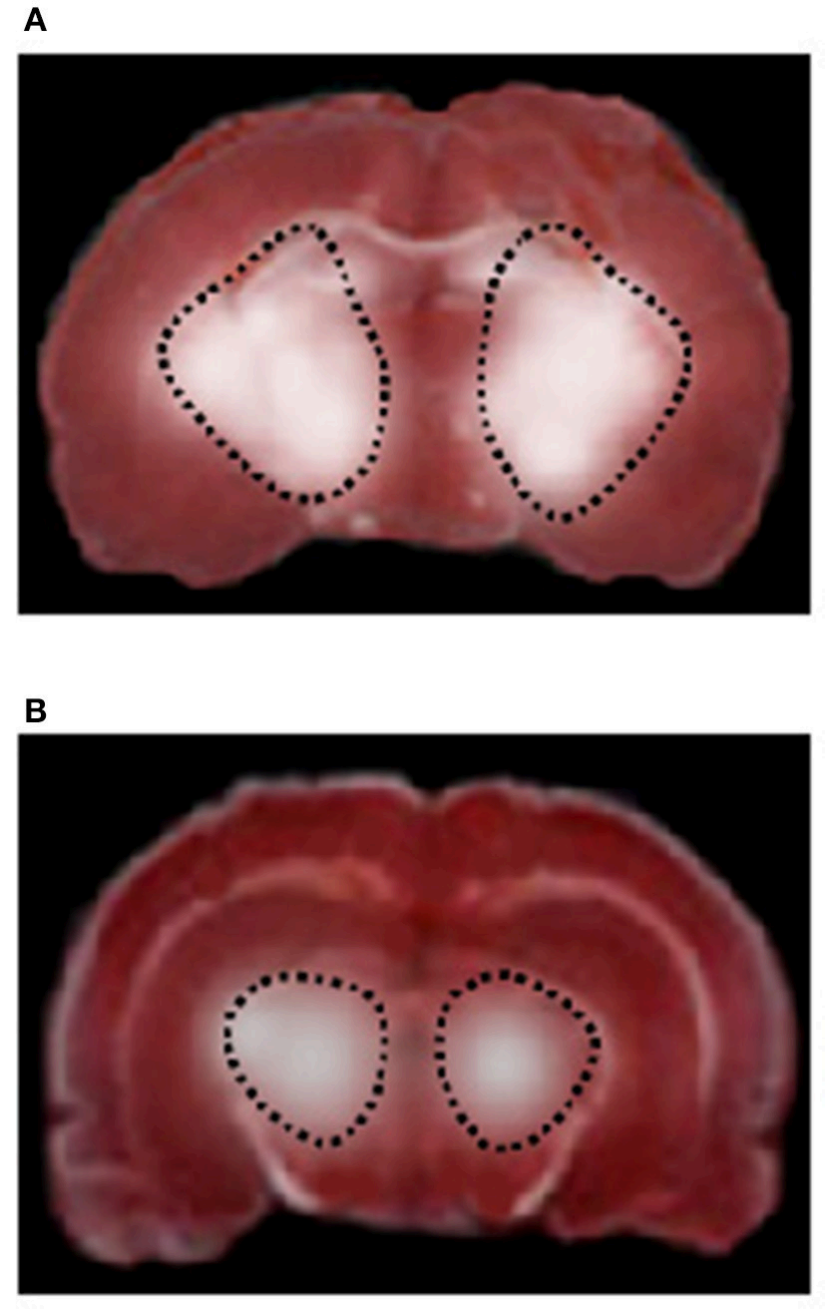
TTC staining of coronal brain slices from a rat of RF group **(A)** and higher dosage cyanidin-treated subgroup **(B)** after 30 min CBFD and 60 min CBFR. The lesion in the striatum is outlined by the dashed black line.

CBFD and CBFR caused damage in cortex and striatum cerebral tissue of both hemispheres in RF animals, compared to SO-Na subgroup (Figure 4A). Cortex infarct size was 8.6 ± 2.0% (*p* < 0.01 vs. nonhypoperfused cortex), while in the striatum the damage was more marked (striatum infarct size 30.5 ± 3.2%, *p* < 0.01 vs. nonhypoperfused cortex).

Conversely, neuronal damage drastically diminished in rats undergoing CBFD and CBFR treated with cyanidin (Cy_1_ and Cy_2_ subgroups), when compared to the previous subgroups (Figure 4B); in particular, the injury was limited to the striatum (infarct sizes were 25 ± 2% and 16.3 ± 1.8% in rats treated with low or high cyanidin dosage, respectively, *p* < 0.01 vs. hypoperfused striatum).

## Discussion

The present data indicate that cyanidin, a polyphenol widely diffused in nature, was able to counteract oxidative stress and microvascular changes induced by 30 min of cerebral blood flow decrease, caused by bilateral occlusion of common carotid arteries, and 60 min of cerebral blood flow recovery. These data are the first evidence of ROS reduction in rat pial networks during cyanidin administration under conditions of reduced blood flow and reperfusion. We characterized the pial microcirculation by fluorescence microscopy to study microvascular responses after acute injury. The alterations caused by the reduction in cerebral blood flow and subsequent reperfusion were characterized by arteriolar constriction, venular permeability increase, leukocyte adhesion to venular walls and capillary rarefaction, as previously reported (Lapi et al., 2016; Mastantuono et al., 2016). Moreover, we observed a dramatic increase in ROS production, as detected by DCFH-DA assay.

Interestingly, cyanidin protected rat pial microcirculation during the decrease in cerebral blood flow and consequent reperfusion in a dose-related manner. We chose to analyze cyanidin effects on arteriolar responses, because these vessels were mainly involved in the regulation of tissue perfusion (Martinez-Lemus, 2012). At the end of the restriction in blood flow, cyanidin prevented the decrease in diameter of order 2 arterioles, compared with RF group. Moreover, at the end of the time-period of blood flow recovery, higher dosage cyanidin induced a vasodilation by 40.5% of baseline in order 2 arterioles. These vasodilatory effects were accompanied by preservation of capillary perfusion, significant decrease in venular leakage as well as in leukocyte adhesion to venular walls. The latter effects may be related to the reduction in ROS formation induced by cyanidin, because ROS have been shown to play a crucial role in the regulation of vessel wall permeability and adhesion of leukocyte to venular walls (Accetta et al., 2016; Mondola et al., 2016). It is worth noting, indeed, that ROS formation was marked in RF group, but resulted lower in cyanidin-treated animals.

Arteriolar vasodilation was counteracted by L-NIO, an inhibitor of the endothelial isoform of nitric oxide synthase (NOS), injected 10 min before cyanidin; therefore, it is possible to hypothesize that cyanidin vasodilatory properties may be due to NO release from vascular endothelial cells. These data are in agreement with previous studies indicating that this polyphenol was able to enhance the release of NO and other vasodilating factors, improving the endothelial-dependent vasodilation (Sivasinprasasn et al., 2016). Interestingly, cyanidin protective effects on leakage were not abolished by L-NIO infusion, suggesting that this polyphenol is able to protect the blood brain barrier integrity mainly through its scavenger activity rather than through its vasodilatory properties.

The biochemical properties of cyanidin have been widely studied; this molecule showed several antioxidant and anti-inflammatory activities (Olivas-Aguirre et al., 2016). In particular, the highest ORAC (oxygen radical absorbance capacity) activity has been demonstrated for cyanidin, compared to other anthocyanins (Zheng and Wang, 2003). Moreover, this polyphenol down-regulates plasminogen activator inhibitor-1 and pro-inflammatory cytokine IL-6, ameliorating human adipocytokine profile (Tsuda et al., 2006). It is interesting to note that isolated rat hearts are protected by oxidative stress, increased in several cardiovascular diseases, after treatment with cyanidin (Ziberna et al., 2012) as well as rat hepatic I/R damage is decreased by cyanidin (Tsuda et al., 1999). In the present study, cyanidin showed a strong antioxidant activity reducing ROS production, but at the same time was able to decrease adhesion of leukocytes to vessel walls blunting the damage induced by leukocyte activation, a mechanism effective in promoting ROS generation. Therefore, the decrease in leukocyte sticking to vessels walls and the reduction in ROS generation merged and reduced the effects of ROS on vessel wall leakage. These effects were effective in preventing marked microvascular damage, avoiding the disruption of blood-brain barrier and tissue edema. All together these vasodilatory and antioxidant properties preserved cerebral perfusion and prevented neuronal loss at the end of blood flow recovery in cyanidin-treated animals. Consequently, there was a reduction of the infarct size in cortical and striatal zones, compared to the rats subjected to decrease in cerebral blood flow and subsequent recovery.

Our data are in agreement with previous observations by Di Giacomo et al. who have observed the effects of Cyanidin-3-O-glucoside injection before the bilateral common carotid artery occlusion and during reperfusion. Their data indicate that cyanidin is able to reduce the lipid hydroperoxides and the expression of neuronal and inducible NOS and to increase the expression in endothelial nitric oxide synthase (eNOS) (Di Giacomo et al., 2012).

In conclusion, our data are the first evidence in an *in vivo* study that rat pial microcirculation was protected against different mechanisms of damage: cyanidin was able to induce arteriolar dilation, to reduce oxidative stress and prevent neuronal loss. All these activities resulted in the protection of cerebral perfusion, blood brain barrier integrity and brain function. Therefore, cyanidin appears to be useful in counteracting ROS generation in brain circulation and to protect cerebral tissues.

## Author contributions

Conceived and designed the experiments: TM, AC, and DL. Performed the experiments and the animal treatments: TM, MD, MC, LB, NS, AC, and DL. Analyzed the data: TM, MD, MC, LB, EM, GN, AC, and DL. Wrote the paper: TM, MD, MC, AC, and DL.

### Conflict of interest statement

The authors declare that the research was conducted in the absence of any commercial or financial relationships that could be construed as a potential conflict of interest.

## References

[B1] Accetta R. Damiano S. Morano A. Mondola P. Paternò R. Avvedimento E. V. (2016). Reactive oxygen species derived from NOX3 and NOX5 drive differentiation of human oligodendrocytes. Front. Cell Neurosci. 10:146 10.3389/fncel.2016.00146 PMC4889614 27313511

[B2] Bederson J. B. Pitts L. H. Germano S. M. Nishimura M. C. Davis R. L. Bartkowski H. M. (1986). Evaluation of 2,3,5-triphenyltetrazolium chloride as a stain for detection and quantification of experimental cerebral infarction in rats. Stroke 17, 1304–1308. 2433817 10.1161/01.str.17.6.1304

[B3] Di Giacomo C. Acquaviva R. Santangelo R. Sorrenti V. Vanella L. Li Volti G. (2012). Effect of treatment with cyanidin-3-O-β-D-glucoside on rat ischemic/reperfusion brain damage. Evid. Based Comp. Alternat. Med. 2012:285750 10.1155/2012/285750 PMC3449154 23008739

[B4] Fratantonio D. Cimino F. Molonia M. S. Ferrari D. Saija A. Virgili F.. (2016). Cyanidin-3-O-glucoside ameliorates palmitate-induced insulin resistance by modulating IRS-1 phosphorylation and release of endothelial derived vasoactive factors. Biochim. Biophys. Acta 1862, 351–357. 10.1016/j.bbalip.2016.12.008 28011403

[B5] Galvano F. La Fauci L. Lazzarino G. Fogliano V. Ritieni A. Ciappellano S. (2004). Cyanidins: metabolism and biological properties. J. Nutr. Biochem. 15, 2–11. 10.1016/j.jnutbio.2003.07.004 14711454

[B6] Hudetz A. G. Fehér G. Weigle C. G. Knuese D. E. Kampine J. P. (1985). Video microscopy of cerebrocortical capillary flow: response to hypotension and intracranial hypertension. Am. J. Physiol. 268, H2202–H2210. 7611470 10.1152/ajpheart.1995.268.6.H2202

[B7] Isaak C. K. Wang P. Prashar S. O K. Brown D. C. Debnath S. C.. (2017). Supplementing diet with Manitoba lingonberry juice reduces kidney ischemia-reperfusion injury. J. Sci. Food Agric. 97, 3065–3076. 10.1002/jsfa.8200 28074603

[B8] Jakesevic M. Xu J. Aaby K. Jeppsson B. Ahrn,é S. Molin G. (2013). Effects of bilberry (*Vaccinium myrtillus*) in combination with lactic acid bacteria on intestinal oxidative stress induced by ischemia-reperfusion in mouse. J. Agric. Food Chem. 61, 3468–3478. 10.1021/jf400203h 23488931

[B9] Kassab G. S. Rider C. A. Tang N. J. Fung Y. C. (1993). Morphometry of pig coronary arterial trees. Am. J. Physiol. 265, H350–H365. 8342652 10.1152/ajpheart.1993.265.1.H350

[B10] Lapi D. Chiurazzi M. Di Maro M. Mastantuono T. Battiloro L. Sabatino L. (2016). Malvidin's effects on rat pial microvascular permeability changes due to hypoperfusion and reperfusion injury. Front. Cell Neurosci. 10:153 10.3389/fncel.2016.00153 PMC4927580 27445688

[B11] Lapi D. Marchiafava P. L. Colantuoni A. (2008). Geometric Characteristics of arterial network of rat pial microcirculation. J. Vasc. Res. 45, 69–77. 10.1159/000109078 17901708

[B12] Lapi D. Mastantuono T. Sapio D. Paterni M. Colantuoni A. (2013). Pial Microvascular responses induced by transient bilateral common carotid artery occlusion in Zucker rats. Clin. Hemorheol. Microcirc. 54, 415–429. 10.3233/CH-131763 24002119

[B13] Lapi D. Vagnani S. Pignataro G. Esposito E. Paterni M. Colantuoni A. (2012). Protective effects of quercetin on rat pial microvascular changes during transient bilateral common carotid artery oclusion and reperfusion. Front. Physiol. 32, 1–12. 10.3389/phys.2012.00032 PMC3290798 22403549

[B14] Martinez-Lemus L. A. (2012). The dynamic structure of arterioles. Basic Clin. Pharmacol. Toxicol. 110, 5–11. 10.1111/j.1742-7843.2011.00813.x 21989114 PMC4435689

[B15] Mastantuono T. Starita N. Sapio D. D'Avanzo S. A. Di Maro M. Muscariello E.. (2016). The effects of *Vaccinium myrtillus* extract on hamster pial microcirculation during hypoperfusion-reperfusion injury. PLoS ONE 11:e0150659. 10.1371/journal.pone.0150659 27070318 PMC4829249

[B16] Mondola P. Damiano S. Sasso A. Santillo M. (2016). The Cu, Zn superoxide dismutase: not only a dismutase enzyme. Front. Physiol. 7:594. 10.3389/fphys.2016.00594 27965593 PMC5126113

[B17] Moreau P. Takasa H. Küng C. F. van Rooijen M. M. Schaffner T. Lüscher T. F. (1995). Structure and function of the rat asilar artery during chronic nitric oxide synthase inhibition. Stroke 26, 1922–1928. 7570749 10.1161/01.str.26.10.1922

[B18] Morii S. Ngai A. C. Winn H. R. (1986). Reactivity of rat pial arterioles and venules to adenosine and carbon dioxine: with detailed description of the closed cranial window technique in rats. J. Cereb. Blood Flow Metab. 6, 34–41. 10.1038/jcbfm.1986.5 3080442

[B19] Ngai A. C. Ko K. R. Morii S. Winn H. R. (1988). Effect of sciatic nerve stimulation on pial arterioles in rats. Am. J. Physiol. 254, H133–H139. 3337250 10.1152/ajpheart.1988.254.1.H133

[B20] Olivas-Aguirre F. J. Rodrigo-García J. Martínez-Ruiz N. D. Cárdenas-Robles A. I. Mendoza-Díaz S. O. Álvarez-Parrilla E. (2016). Cyanidin-3-O-glucoside: physical-chemistry, foodomics and health effects. Molecules 21:E1264 10.3390/molecules21091264 27657039 PMC6273591

[B21] Quintieri A. M. Baldino N. Filice E. Seta L. Vitetti A. Tota B.. (2013). Malvidin, a red wine polyphenol, modulates mammalian myocardial and coronary performance and protects the heart against ischemia/reperfusion injury. J. Nutr. Biochem. 24, 1221–1231. 10.1016/j.jnutbio.2012.09.006 23266283

[B22] Serraino I. Dugo L. Dugo P. Mondello L. Mazzon E. Dugo G.. (2003). Protective effects of cyanidin-3-O-glucoside from blackberry extract against peroxynitrite-induced endothelial dysfunction and vascular failure. Life Sci. 73, 1097–1114. 10.1016/S0024-3205(03)00356-4 12818719

[B23] Sivasinprasasn S. Pantan R. Thummayot S. Tocharus J. Suksamrarn A. Tocharus C. (2016). Cyanidin-3-glucoside attenuates angiotensin II-induced oxidative stress and inflammation in vascular endothelial cells. Chem Biol Interact. 260, 67–74. 10.1016/j.cbi.2016.10.022 27983965

[B24] Tsuda T. Horio F. Kitoh J. Osawa T. (1999). Protective Effects of Dietary Cyanidin 3-O-β-d-Glucoside on Liver Ischemia–Reperfusion Injury in Rats. Arch. Biochem. Biophys. 368, 361–366. 10441388 10.1006/abbi.1999.1311

[B25] Tsuda T. Ueno Y. Yoshikawa T. Kojo H. Osawa T. (2006). Microarray profiling of gene expression in human adipocytes in response to anthocyanins. Biochem. Pharmacol. 71, 1184–1197. 10.1016/j.bcp.2005.12.042 16483547

[B26] Wang H. Joseph J. A. (1999). Quantifying cellular oxidative stress by dichlorofluorescein assay using microplate reader. Free Radic Biol Med. 27, 612–616. 10490282 10.1016/s0891-5849(99)00107-0

[B27] Watanabe S. (1998). *In vivo* fluorometric measurement of cerebral oxidative stress using 2'-7'- dichlorofluorescein (DCF). Keio J Med. 47, 92–98. 9659819 10.2302/kjm.47.92

[B28] Zheng W. Wang S.Y. (2003). Oxygen radical absorbing capacity of phenolics in blueberries, cranberries, chokeberries, and lingonberries. J. Agric. Food Chem. 51, 502–509. 10.1021/jf020728u 12517117

[B29] Ziberna L. Tramer F. Moze S. Vrhovsek U. Mattivi F. Passamonti S. (2012). Transport and bioactivity of cyaniding 3-glucoside into the vascular endothelium. Free Radic. Biol. Med. 52, 1750–1759. 10.1016/j.freeradbiomed.2012.02.027 22387282

